# Development of a High-Throughput Assay for Screening of γ-Secretase Inhibitor with Endogenous Human, Mouse or Drosophila γ-Secretase

**DOI:** 10.3390/molecules14093589

**Published:** 2009-09-14

**Authors:** Lie-Feng Wang, Ru Zhang, Xin Xie

**Affiliations:** 1 Laboratory of Receptor-based Bio-medicine, School of Life Sciences and Technology, Tongji University, Shanghai 200092, China; E-mails: wlf0709@yahoo.com.cn (L-F.W.); ru.zhang@tongji.edu.cn (R.Z.); 2 State Key Laboratory of Drug Research, The National Center for Drug Screening, Shanghai Institute of Materia Medica, Chinese Academy of Sciences, Shanghai 201203, China

**Keywords:** γ-secretase inhibitor, fluorogenic substrate, high throughput screening

## Abstract

Selective lowering of amyloid-β levels with small-molecule γ-secretase inhibitors is a promising therapeutic approach for Alzheimer’s disease. In this work, we developed a high throughput assay for screening of γ-secretase inhibitors with endogenous γ-secretase and a fluorogenic substrate. The IC_50_ values of known γ-secretase inhibitors generated with this method were comparable with reported values obtained by other methods. The assay was optimized and applied to a small-scale screening of 1,280 compounds. The discovery of several new inhibitors warrants further investigation. This assay was also proven to be easily adopted to test compounds for drosophila and mouse γ-secretase, which could be very useful to assess compounds activity against γ-secretase from different species before the *in vivo* test in animal models.

## 1. Introduction

Alzheimer’s disease (AD) is the most common neurodegenerative disorder of the central nervous system. Amyloid plaques, mainly composed of the β-amyloid protein (Aβ), are a major characteristic of Alzheimer neuropathology and considered to be the primary cause of the disease [1]. Sequential cleavage of amyloid precursor protein (APP), a ubiquitously expressed type I membrane glycoprotein, by β-secretase and by γ-secretase, generates Aβ. The Aβ peptides generated in the proteolytic pathways vary in length from 38 to 43 amino acids with Aβ_40_ as the predominant secreted Aβ species. The longer form (Aβ_42_) is a minority of all Aβ peptides, but being much more prone to aggregation, it is the major species in the diffuse plaques that represent the earliest stage of Aβ deposition [2].

The γ-secretase complex is composed of at least four membrane proteins: presenilin (PS), nicastrin (NCT), APH-1 and PEN2 [3]. Most Familial Alzheimer’s disease (FAD)-causing mutations are found to localize on the two PS genes (PS1 and PS2) and correlate with the selective elevation of Aβ42 versus Aβ40 [4]. The pivotal role of γ-secretase in AD pathogenesis leads to drug development based on this target. The first reported *in vivo* testing of a γ-secretase inhibitor involved the dipeptidic compound DAPT, developed by ELAN and Eli Lilly. This compound potently inhibited Aβ production in human primary neuronal culture and HEK 293 cells [5]. L-685,458, an aspartyl protease transition state mimic, reduced both Aβ40 and Aβ42 peptide formation in SH-SY5Y, Neuro2a and CHO cells [6]. A large Phase III study is currently underway to examine the therapeutic effect of a new γ-secretase inhibitor semagacestat (LY450139). And a number of other candidate γ-secretase inhibitors are entering clinical trials [7,8].

Currently most of the methods for screening of γ-secretase inhibitors are based on cellular Aβ detection. These methods usually need cell lines that over-express human APP and the costly sandwich ELISA method of detection [5,6,9]. To search for new γ-secretase inhibitors, we have developed and validated a high-throughput screening (HTS) assay utilizing a specific fluorogenic substrate and the endogenously expressed γ-secretase in HEK293T cells. This assay was also proved to be easily adopted to test compounds on drosophila and mouse γ-secretase, which could be very useful to assess compounds activity against γ-secretase from different species before the *in vivo* test in animal models. This assay was also applied to screen the LOPAC library containing 1,280 synthetic compounds. Eight compounds with γ-secretase modulation activity were identified.

## 2. Results and Discussion

### 2.1. Assay optimization

**Figure 1:**
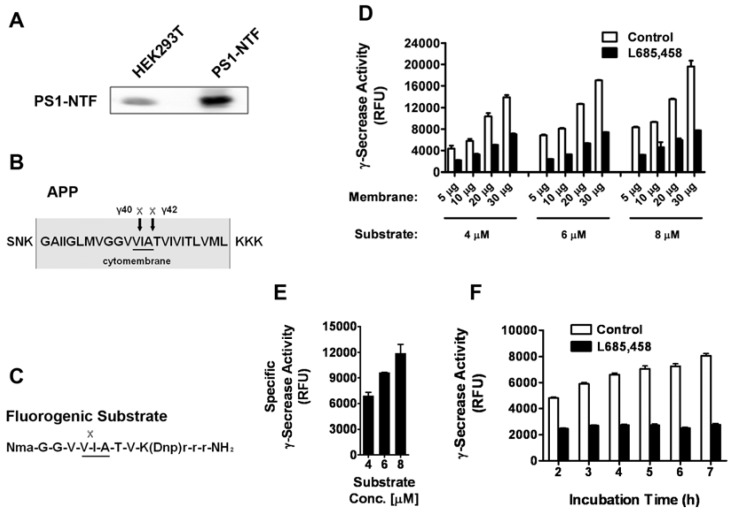
Assay optimization. A. Western blot analysis of the expression of PS-1 in HEK293T cells. HEK293T cells were transfected with a plasmid encoding the N-terminal fragment of human PS1 (PS1-NTF) or not (HEK293T), and the samples were subjected to western blot assay. B. The sequence of APP with the γ-secretase cleavage sites. C. The sequence of the fluorogenic substrate. D. Various amount of membrane proteins were incubated with 4, 6 or 8 μM of fluorogenic substrate for 5 h with or without the presence of 1 μM L-685,458. The γ-secretase activity was presented as the relative fluorescent unit (RFU). E. The specific γ-secrease activity increases with the increase of substrate concentration. F. 10 μg of membrane proteins were incubated with 6 μM of substrate for various periods of time, and γ-secretase activity were measured. Data was shown as Mean±SEM of at least three independent experiments. (n ≥ 3).

HEK293T cells have been reported to express the active γ-secretase complex [10,11] and our Western blot analysis also indicated the endogenous expression of PS-1, the catalytic subunit of γ-secretase (Figure 1A). This cell line provides us an easy way to collect large quantities of human γ-secretase containing membranes. An intra-molecularly quenched fluorogenic substrate [12] containing the C-terminal amino acid sequence of APP (an endogenous substrate of γ-secretase, Figure 1B and C) that is recognized and cleaved by γ-secretase was utilized. Membrane was prepared from HEK293T cells and solubilized γ-secretase was generated with CHAPSO detergent containing buffer as previously described [4,13]. γ-Secretase mediated cleavage of the substrate was monitored by measuring fluorescence after incubating solubilized membrane at 37°C in the absence or presence of L-685,458, a known specific transition state analogue inhibitor of γ-secretase. Various experimental conditions, including membrane amount, substrate concentration and incubation time, were tested. We found with the increase of membrane amount and substrate concentration, the total fluorescent signal increased dramatically (Figure 1D). After subtracting the background fluorescence, we analyzed the specific γ-secrease activity with different substrate concentrations. As shown in Figure 1E, there is a clear dose response with the increase of the substrate concentration. This indicates that the substrate concentrations we used are sub-saturating and the assay should be sensitive to competitive inhibition. The incubation time was found to significantly affect the specific signal with the longer time giving larger signal window (Figure 1F). To be time and cost effective, we eventually decided on the following conditions with membrane protein amount at 10 μg, substrate concentration at 6 μΜ and incubation time at 5 h in 37 °C.

### 2.2. Assay performance 

**Figure 2:**
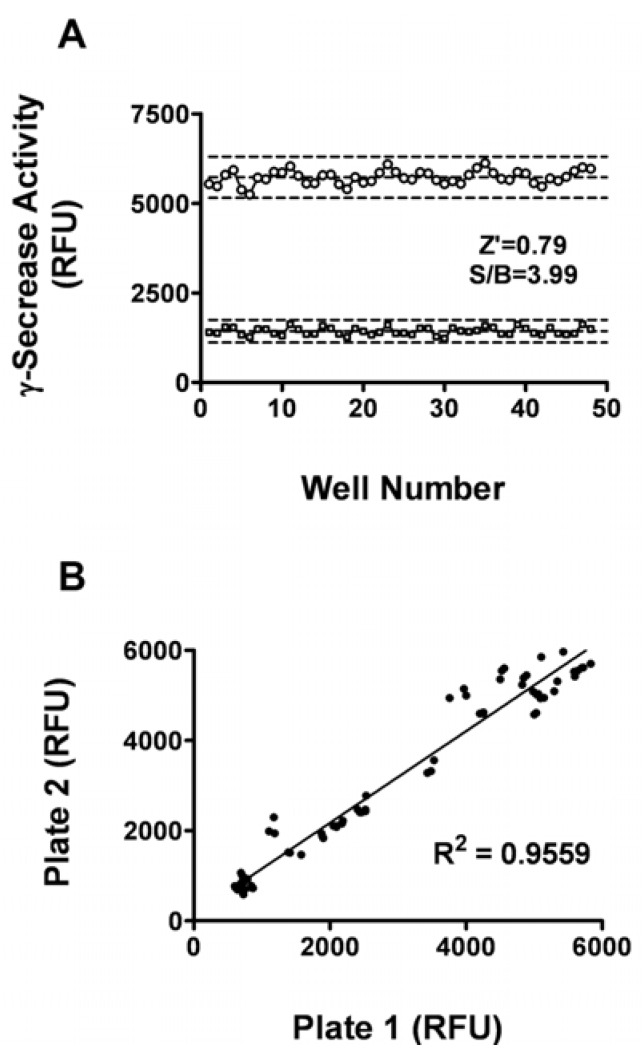
Assay Performance. (A) Z’ factor determination. At the optimized conditions (10 μg membrane proteins, 6 μM substrate, 5 h incubation time), 48 replicates of positive (wells treated with 1% DMSO) and negative signals (wells treated with 1 μM L685,458) were studied. Dashed lines indicate means ± 3 SD of 48 data points. (B) Reproducibility. The corresponding wells from two different 96-well plates were inhibited with same concentration of L685,458. The reproducibility of data from duplicate plates was investigated with linear repression analysis.

The Z’ factor is the normalized three standard deviation window between the negative controls and positive controls. It is widely used for the evaluation of HTS assay qualities. The signal/background (S/B) ratio is another metric used to evaluate the assay window [14]. In general, a Z’ value above 0.5 suggests that an assay is robust enough for HTS. As shown in Figure 2A, the Z’ value for the assay was 0.79 and the S/B ratio was 3.99，indicating that the system was adequately optimized for HTS. Furthermore, to investigate reproducibility between duplicate plates, the corresponding wells from two different 96-well plates were treated with same concentration of L685,458. The data from corresponding wells of different plates were investigated with liner regression analysis. The correlation coefficient was 0.956 (Figure 2B), showing a high degree of reproducibility between duplicate sample plates [15].

### 2.3. The γ-secretase inhibitor activity measurement with human γ-secretase

**Figure 3:**
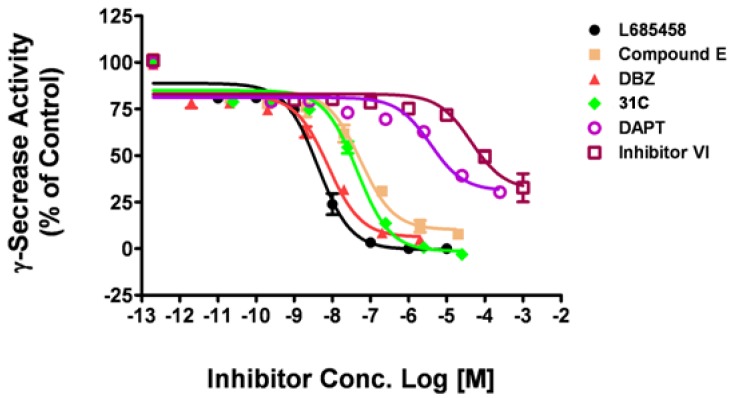
Dose-response curves of known inhibitors against human γ-secretase. Six γ-secretase inhibitors were tested on human γ-secretase obtained from HEK293T cell membrane with the fluorogenic substrate assay. Data are mean ± SEM (n ≥ 3).

Using the optimal assay conditions, we tested a series of known γ-secretase inhibitors of human γ-secretase, including the non-transition state inhibitors, such as DAPT, compound E, and transition state analogs, such as L685,458, DBZ and 31C [16]. L685,458 was used as a positive control and DMSO as a negative control. As shown in Figure 3, three compounds showed inhibitory effect similar to L685,458, with maximal inhibition at 103.1% (31C), 95.2% (DBZ) and 92.3% (Comp E), respectively. The other two compounds displayed partial inhibitory activity with maximal inhibition at 69.7% (DAPT) and 67.3% (Inhibitor VI, Calbiochem). The IC_50_ values obtained with this fluorogenic substrate assay were listed in Table 1. The IC_50_ of L685,485, DBZ and DAPT matched quite well with previously reported values. For 31C, this assay is approximately 10 times more sensitive than the reported one. But for compound E and Inhibitor VI, this assay is about 20 times less sensitive than the reported ones. 

### 2.4. The γ-secretase inhibitor activity measurement with drosophila and mouse γ-secretase

**Figure 4:**
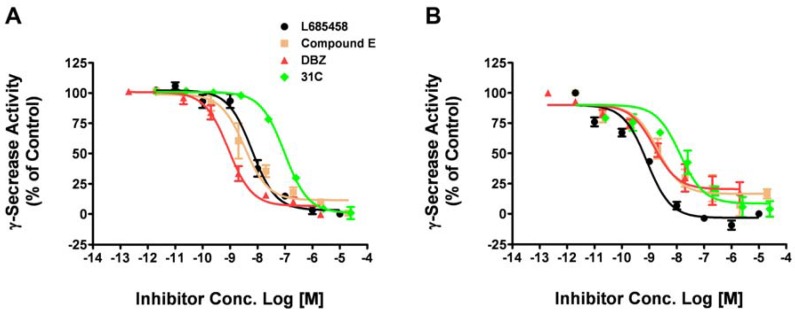
Dose-response curves of known inhibitors against drosophila and mouse γ-secretase. Four γ-secretase inhibitors were tested on drosophila γ-secretase obtained from S2 cell membrane (A) and mouse γ-secretase from mouse cortex membrane with the fluorogenic substrate assay. Data are mean ± SEM (n = 3).

Drosophila has provided us a useful model animal system to study fundamental pathways in physiological and pathological conditions. It has also been applied to elucidate mechanisms of human neurodegenerative disorders including Alzheimer's diseases [22,23]. Transgenic mice have also been widely used to evaluate the therapeutic effects of compounds and drug candidates for Alzheimer's diseases [24,25]. Due to the time consuming nature of these in vivo studies, it would be very useful to assess compound activity against γ-secretase from different species *in vitro* before the animal tests. So we tested whether or not our fluorogenic substrate assay could be applied to drosophila or mouse γ-secretase. The membrane containing drosophila or mouse γ-secretase was isolated from S2 cells or mouse cortex, respectively. Using the optimal assay conditions, we tested four γ-secretase inhibitors (L685,458, Compound E, DBZ, 31C) on drosophila γ-secretase (Figure 4A). The IC_50_ values obtained with drosophila γ-secretase is comparable with human γ-secretase (Table 2).

We then tested these γ-secretase inhibitors on mouse γ-secretase either (Figure 4B). The IC_50_ values obtained with mouse γ-secretase is in the same range as human and drosophila γ-secretase (Table 2). These results indicate that this assay can be easily adapted to test compounds in different species and are a useful assay to assess compounds activity against γ-secretase before the in vivo test in different animal models.

### 2.5. HTS campaign

**Figure 5:**
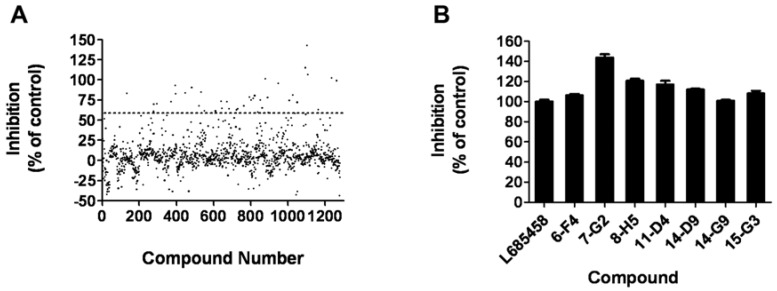
High-throughput screening of 1280 compounds. (A) Result of the primary screening of 1280 compounds in duplicate setup with HEK293T membrane. Compounds with more than 60% inhibition of the γ-secretase were further tested. (B) In the secondary screening with a triplet setup, seven compounds displayed consistant inhibitory effect compare to the known γ-secretase inhibitor L685,458.

The assay was applied to a small-scale screening of a compound library consisting of 1280 compounds with membrane isolated from HEK293T cells, which contains the human γ-secretase complex. In the primary screening, compounds were tested at 10 μM in a duplicate setup. Thirty one hits (2.4%) showing more than 60% inhibition of the γ-secretase were identified (Figure 5A). Secondary screening was carried out to further confirm the hits in a triplicate setup using L685,458 as a positive control (Figure 5B). Finally, seven compounds displaying consistent inhibitory effects were picked out and will be further characterized.

## 3. Experimental 

### 3.1. Reagents

The fluorogenic substrate, γ-secretase inhibitors and CHAPSO were purchased from Merck KGaA (Darmstadt, Germany). Cell culture medium and fetal bovine serum were bought from Invitrogen (Carlsbad, CA, USA). Tris base was purchased from Amresco (Solon, OH, USA). Complete^TM^ protease inhibitor cocktail was purchased from Roche Applied Science (Basel, Switzerland). Other reagents and solvents used in the experiments were of analytical grade.

### 3.2. Cell culture 

HEK293T cells obtained from ATCC (Manassas, VA, USA) were maintained in high-glucose Dulbecco’s modified Eagle’s medium(HG-DMEM) supplemented with 10% fetal bovine serum, 100 mg/L penicillin and 100 mg/L streptomycin at 37 °C in a humidified atmosphere of 5% CO_2_. Cells were trypsinized and seeded onto 10 cm plates at proper density for 48 h before harvesting. Drosophila Schneider 2 (S2) cells obtained from Invitrogen (Carlsbad, CA, USA) were maintained in Schneider’s Drosophila medium containing 10% fetal bovine serum in a 28 °C incubator_._

### 3.3. Animals

Adult C57BL/6 mice were purchased from Shanghai Laboratory Animal Center, Chinese Academy of Sciences one week prior to surgery. The animals were housed under a 12-h light/dark cycle in a climate-controlled room with food and water available *ad libitum.* All experimental procedures were performed in accordance with the National Institutes of Heath Guide for the Care and Use of Laboratory Animals.

### 3.4. Membrane protein preparation from cells and mouse brain

All procedures were carried out at 4 °C. HEK 293T or S2 cells were washed with ice-cold PBS by centrifugation at 500 g for 5 min and the pellets were homogenized with a Dounce homogenizer (pestle B, 15 strokes) in lysis buffer contained 5 mM Tris-HCl (pH 7.4), 5 mM EDTA, 5 mM EGTA and Complete^TM^ protease inhibitor cocktail. Mouse cortex was homogenized with 15 stokes of pestle A in the same lysis buffer. The homogenates were centrifuged at 800 g for 10 min to remove nuclei and large debris. The supernatants were centrifuged at 25,000 g for 1 h at 4 °C and the membrane pellets were resuspended in reaction buffer containing 50 mM Tris-HCl (pH 6.8), 2 mM EDTA, 150 mM KCl and Complete^TM^ protease inhibitor cocktail. After measuring the protein concentration, the membranes were diluted to 1 mg/ml in 50 mM Tris-HCl, pH 6.8, 2 mM EDTA, 150 mM KCl, 0.25% CHAPSO.

### 3.5. Fluorogenic substrate assay

For assay optimization, aliquots of membrane protein (5~30 μg) was added to each well of a 96-well plate with various concentrations of fluorogenic substrate (4~8 μM). Different concentrations of compounds were added and the final reaction system was adjusted to 100 μL/well with the reaction buffer. The plate was incubated at 37 °C in a humidified atmosphere for indicated time periods. Fluorescence was measured using a BIOTEK FLX800 microplate reader with the excitation wavelength at 355 nm and the emission wavelength at 440 nm. The activity of γ-secretase was presented as the relative fluorescent unit (RFU). For compound screening, 1 μM of L685,458 was used as a positive control, and 1% DMSO was used as negative control. The inhibition rate of each compound was calculated with the following equation: Inhibition_Compound_ % = (Average Value_1%DMSO_-Average Value _Compound_)/(Average Value_1%DMSO_-Average Value_L685,458_) × 100%. So the inhibition rate of 1 μM L685,458 is 100% and that of 1% DMSO is 0. The samples showing more than 60% inhibition were considered as “hits” in the screening.

### 3.6. Western blot

HEK293T cells were lysed with SDS loading buffer (50 mmol/L Tris-HCl, 2%SDS, 10% glycerol, 100 mmol/L dithiothreitol, 0.1% bromophenyl blue) and boiled at 95-100 °C for 5 min. Aliquots of proteins were separated with 10% SDS-PAGE and then transferred onto PVDF membrane (BIO-RAD, USA). The membrane was incubated with blocking buffer (20 mmol/L Tris, 137 mmol/L NaCl, 0.1% Tween^®^-20, and 5% non-fat dry milk) for 1h at room temperature. The membrane was then probed with rabbit polyclonal anti-PS1-NTF antibody (529591; Calbiochem, Germany) overnight at 4 °C and then with goat anti-rabbit IgG HRP for 1 h at room temperature. After the final wash with TBST, the membrane was developed on Kodak Imagine station 2000MM (Eastman Kodak, Rochester, NY, USA) using the SuperSignal West Pico chemiluminescent kit according to manufacturer’s instruction (Thermo Scientific, San Jose, CA, USA).

### 3.7. Data analysis

Data were analyzed with GraphPad Prism software (GraphPad, San Diego, CA, USA). Non-linear regression analyses were performed to generate dose-response curves and calculate IC_50_ values, and linear regression was used to analyze data reproducibility. Mean ± SEM was calculated automatically with this software. Z’ factor was calculated by the following equation:

Z’ = 1 － (3SD_＋_＋3SD_－_) / │Ave_＋_ － Ave_－_│

in which SD_＋_ is the standard deviation of positive control, SD_－_is the standard deviation of negative control, Ave_＋_ is the mean value of the positive control, and Ave_－_is the mean value of the negative control [14].

## 4. Conclusions

In this study, we developed a high-throughput assay for screening of γ-secretase inhibitor with endogenously expressing human γ-secretase from HEK293T cell membrane and a fluorogenic substrate. This assay was optimized with the Z’ value at 0.79 and an S/B ratio at 3.99. The IC_50_ values of known γ-secretase inhibitors generated with this method were comparable with reported values obtained by other methods. This assay was also proven to be easily adapted to test compounds for drosophila and mouse γ-secretase inhibitors, which could be very useful to assess compounds’ activity against γ-secretase from different species before the *in vivo* test in animal models. Finally, this assay was applied to a small-scale screening of a compound library consisting of 1,280 compounds, seven compounds with consistent inhibition of γ-secretase were identified and will be further characterized.

## Figures and Tables

**Table 1:** Comparison of IC_50_ values of various compounds obtained by fluorogenic substrate assay and previous reported methods.

Compound	Fluorogenic substrate assay IC_50_ (nM)	Reported IC_50_(nM) / Assay used
L685,458	4.43 ± 0.9691	IC_50_ (Aβ_40_) = 4 nM; IC_50_(Aβ_42_) = 8 nM /immunoblotting assay [9] IC_50_ = 17 ± 8 nM/ HTRF assay [6] IC_50_ = 3 nM / *in vitro* γ-secretase assay [17]
Compound E	53.86 ± 5.312	IC_50_(Aβ_40_) = 0.24 nM; IC_50_(Aβ_42_) = 0.37 nM / immunoblotting assay[9] IC_50_(Aβ_40_) = 0.8 nM; IC_50_(Aβ_42_) = 7 nM / Aβ ELISA assay [18] IC_50_ = 7 nM / *in vitro* γ-secretase assay [17]
DBZ	12.50 ± 5.764	IC_50_ = 1.7 nM / Sup-T1 NICT assay[19]
DAPT	3910 ± 550.7	IC_50_(Aβ_40_) = 1600 nM IC_50_(Aβ_42_) = 4000 nM / Aβ ELISA assay [18]
31C	53.48 ± 13.58	IC_50_**(**(Aβ) = 300 nM / *In vivo* γ-secretase assay [20]
Inhibitor VI	44457 ± 25568	IC_50_ (Aβ_1-42_) = 1800 nM [21]

**Table 2:** Comparison of the IC_50_ values of various compounds obtained with human, drosophila and mouse γ-secretase.

Compound	human γ-secretase IC_50_ (nM)	drosophila γ-secretase IC_50_ (nM)	mouse γ-secretase IC_50_ (nM)
L685,458	4.43 ± 0.9691	10.35 ± 1.657	0.9759 ± 0.1467
Compound E	53.86 ± 5.312	40.55 ± 6.997	2.398 ± 0.6046
DBZ	12.50 ± 5.764	6.970 ± 1.984	1.446 ± 0.4350
31C	53.48 ± 13.58	0.9328 ± 0.1690	20.69 ± 5.242
